# Hepatoprotective Effect of Citral on Acetaminophen-Induced Liver Toxicity in Mice

**DOI:** 10.1155/2017/1796209

**Published:** 2017-06-22

**Authors:** Nancy Sayuri Uchida, Saulo Euclides Silva-Filho, Gabriel Fernando Esteves Cardia, Edivaldo Cremer, Francielli Maria de Souza Silva-Comar, Expedito Leite Silva, Ciomar Aparecida Bersani-Amado, Roberto Kenji Nakamura Cuman

**Affiliations:** ^1^Department of Pharmacology and Therapeutics, State University of Maringá, Avenida Colombo 5790, 870020-900 Maringá, PR, Brazil; ^2^Department of Chemistry, State University of Maringá, 87020-900 Maringá, PR, Brazil

## Abstract

High doses of acetaminophen (APAP) lead to acute liver damage. In this study, we evaluated the effects of citral in a murine model of hepatotoxicity induced by APAP. The liver function markers alanine aminotransferase (ALT), aspartate aminotransferase (AST), alkaline phosphatase (ALP), and gamma-glutamyl transferase (*γ*GT) were determined to evaluate the hepatoprotective effects of citral. The livers were used to determine myeloperoxidase (MPO) activity and nitric oxide (NO) production and in histological analysis. The effect of citral on leukocyte migration and antioxidant activity was evaluated in vitro. Citral pretreatment decreased significantly the levels of ALT, AST, ALP, and *γ*GT, MPO activity, and NO production. The histopathological analysis showed an improvement of hepatic lesions in mice after citral pretreatment. Citral inhibited neutrophil migration and exhibited antioxidant activity. Our results suggest that citral protects the liver against liver toxicity induced by APAP.

## 1. Introduction

Drug-induced liver injury is a significant clinical problem worldwide [1]. Most drug-induced liver injury and acute liver failure occur due to either accidental or intentional overdose of acetaminophen (N-acetyl-p-aminophenol, paracetamol, APAP) [2]. APAP is an antipyretic and analgesic drug used widely in clinics. When used at therapeutic doses, APAP is metabolized by glucuronidation or sulfation by the cytochrome p450 system into the reactive metabolite N-acetyl-p-benzoquinone imine (NAPQI). Under normal circumstances, NAPQI is rapidly converted to nontoxic metabolites by glutathione (GSH). However, at large doses of APAP, NAPQI levels increase and may react with hepatic proteins, resulting in liver injury [3, 4]. Because of its dose-dependent toxicity, APAP-induced hepatic damage can be studied in animal models and most mechanisms are translatable to humans [2].

APAP-induced hepatotoxicity has been a significant issue for several years and different strategies have been studied, including the use of natural compounds with hepatoprotective effects [5, 6].

Natural products are attracting the interest of many researchers to investigate their potential as drugs for the treatment of various diseases. Furthermore, there are many bioactive substances that are synthesized from constituents of essential oils (mixture of volatile and natural substances) that have some pharmacological activities [7]. In this context, monoterpene citral, an isomeric mixture of neral and geranial, a component of lemongrass oil, has been reported to have many biological activities such as antibacterial and anti-inflammatory activities [8–10]. However, the protective effect of citral on APAP-toxicity remains unclear. Thus, the aim of the present study was to evaluate the effect of citral in hepatotoxicity induced by APAP.

## 2. Materials and Methods

### 2.1. Plant Material and Constituent of Essential Oils

The constituent citral was isolated from lemongrass essential oil as fractions of hydrodistilled oil and was identified by GC-MS and NMR as previously described [11].

### 2.2. Animals

Male* Swiss* mice (30–40 g) were provided by the Central Animal House of the State University. The animals were maintained under controlled environmental conditions of temperature (22 ± 2°C) and 12/12 h light/dark cycle. Prior to the experiments, the animals fasted overnight, with water provided ad libitum. The experimental protocols were approved by the Ethical Committee in Animal Experimentation of the State University of Maringá (CEAE/UEM 084/2014).

### 2.3. Cell Viability Analysis

The MTT (3-[4 5-dimethylthiazol-2-yl]-2,5-diphenyl-2H-tetrazolium bromide) assay is based on the mitochondrial enzyme reduction of tetrazolium dye to detect and determine cell viability. Leukocytes were obtained from the peritoneal cavity of mice 4 h after zymosan injection (1 mg/cavity, i.p.). Briefly, the cells were plated at a density of 5 × 10^5^ cells/well in a volume of 100 *μ*L RPMI 1640 medium supplemented with 10% fetal bovine serum and 100 U/mL penicillin + 100 *μ*g/mL streptomycin in 96-well plates. The cells were incubated with varying concentrations of citral (3, 10, 30, and 90 *μ*g/mL) or vehicle (0.1% Tween 80 solution, used as control), at 37°C in 5% CO_2_ for 90 min followed by the addition of 10 *μ*L MTT (5 mg/mL) stock solution to each well. After 2 h of incubation at 37°C, 150 *μ*L of the supernatant was removed, and 100 *μ*L dimethyl sulfoxide was added to each well. The cells were incubated at 25°C for a further 10 min, and absorbance was measured using a Biochrom Asys Expert plus microplate reader at a wavelength of 540 nm. The values obtained of the blank (RPMI 1640) wells were subtracted from each well of treated and control cells. Viability was determined using the equation

(1) $$\begin{array}{c} \text{Viability }\left(\;\%\;\right)=\frac{\left(At-Ab\right)}{\left(Ac-Ab\right)}\times 100, \end{array}$$

where At, Ab, and Ac are the absorbance of treated cells, blank, and control, respectively.

### 2.4. DPPH Radical Scavenging

Free radical scavenging capacity (RSC) was evaluated by measuring the 2,2-diphenyl-1-picrylhydrazyl- (DPPH-) scavenging activity of citral. The DPPH assay was performed as previously described [12] with minor modifications. The samples 125–5000 *μ*g/mL were mixed with 1 mL of 25 mM of DPPH^•^ solution, with the addition of 95% methanol to a final volume of 4 mL. The absorbance of the resulting solutions and blank (i.e., with the same chemicals, with the exception of the sample) was recorded against ascorbic acid (Chem Cruz; used as a positive control) after 30 min at room temperature. For each sample, four replicates were recorded. The disappearance of DPPH^•^ was measured spectrophotometrically at 515 nm using a Beckman DU-65 spectrophotometer. The percentage of the RSC was calculated using the following equation: RSC (%) = 100 × (*A*_blank_ − *A*_sample_/*A*_blank_). The IC_50_ value, representing the concentration of the citral that caused 50% RSC inhibition, was determined by linear regression analysis from the obtained RSC values [11, 13].

### 2.5. Treatments

The experimental animals were divided into six groups of five animals each. Firstly, each group received orally during seven days the following treatment: Group I: the mice did not receive any treatment (normal). Group II: the mice received citral vehicle (0.1% Tween 80 solution). Groups III–V: the mice were pretreated with citral at doses of 125, 250, and 500 mg/kg, respectively. Group VI: the mice were pretreated with the hepatoprotective standard drug silymarin (SLM) (200 mg/kg). After this time, the animals fasted for 8 h and then received oral APAP on the seventh day at a dose of 250 mg/kg in Groups II–VI. Group I orally received saline that contained 0.1% Tween 80 solution (APAP vehicle). The stock solution was used as the first concentration of 50 mg/mL and after that was diluted in 0.1% Tween 80 solution to prepare the solutions of 25 and 12.5 mg/mL. After 12 h of APAP administration, serum samples and liver tissue were collected followed by biochemistry and histological analysis [11, 13].

### 2.6. Markers of Liver Function

Blood samples were collected and centrifuged at 3000 ×g for 15 min at 4°C. Biochemical parameters in serum aspartate aminotransferase (AST), alanine aminotransferase (ALT), alkaline phosphatase (ALP), and gamma-glutamyl transferase (*γ*-GT) levels were estimated using the Analyze Gold enzymatic test kit.

### 2.7. Chemotaxis Assay

To evaluate the effects of citral on chemotaxis neutrophils were obtained 4 h after zymosan injection (1 mg/cavity, i.p.) of* Swiss* mice by peritoneal washing with 3 mL of phosphate-buffered saline. The cell number was adjusted to 1 × 10^6^ cells/mL in RPMI 1640 medium that contained 0.1% bovine serum albumin. A chemotaxis assay was performed using a 48-well microchemotaxis plate (Neuro Probe), in which the chambers were separated by a polyvinylpyrrolidone-free polycarbonate membrane (5 *μ*m pore size). The chemoattractant, N-formyl methionyl leucyl phenylalanine (fMLP; 10^−6 ^M), and negative control (vehicle: RPMI 1640) were placed in the lower chamber. A neutrophil suspension (1 × 10^6^ cells/mL) pretreated with citral (1, 3, 10, 30, 60, and 90 *μ*g/mL) for 30 min was then placed in the upper chamber. The cells were allowed to migrate into the membrane for 1 h at 37°C in 5% CO_2_. Following incubation, the membrane was washed in PBS, fixed in methanol, and stained with Instant Prov. The membrane area of each well was scored using light microscopy to count the cells present in five random fields. The results are expressed as the mean number of neutrophils per field and are representative of triplicate measurements from three separate experiments.

### 2.8. Determination of Myeloperoxidase Activity (MPO) on Liver Tissue

The homogenate supernatant of the liver sections was used to determine MPO enzyme activity [14] which were placed in potassium phosphate buffer that contained hexadecyltrimethylammonium bromide in a Potter homogenizer. The homogenate was stirred in a vortex and centrifuged. Ten microliters of the supernatant was added to each well in triplicate in a 96-well microplate. Two hundred microliters of the buffer solution that contained 16.7 mg* O*-dianisidine dihydrochloride (Sigma-Aldrich, St. Louis, MO, USA), 90 mL double-distilled water, 10 mL potassium phosphate buffer, and 50 *μ*L of 1% H_2_O_2_ was added. The enzymatic reaction was stopped by addition of sodium acetate. Enzyme activity was determined by absorbance measured at 450 nm using microplate spectrophotometer (Asys Expert Plus) [11, 13].

### 2.9. Determination of Nitric Oxide (NO) Production

The NO production was determined by the* Griess* method in the supernatant of liver tissue sections, which determines the nitrite production [15]. Fifty microliters of the supernatant was added to each well in triplicate in a 96- well microplate. Sequentially, Griess reagent was added (1% sulfanilamide in 5% phosphoric acid and 0.1% N-(1-naphthyl)ethylenediamine dihydrochloride in* Milli-Q* water) at room temperature. The reading was taken using an ELISA plate reader at a wavelength of 550 nm. NO production was calculated from a standard curve of sodium nitrite [11, 16]. The results were expressed as *μ*M.

### 2.10. Histopathological Analysis

The livers were collected and fixed in 10% formaldehyde solution. Subsequently, they were dehydrated with increasing concentrations of alcohol (80–100%, v/v) and paraffin embedded and sectioned in semiserial at a 5 *μ*m thickness on a Leica rotary microtome (Leica Microsystems, Gladesville, New South Wales, Australia). The sections were stained with hematoxylin and eosin (H&E) and examined for visualization of changes using light microscopy (Olympus BX-41, Tokyo, Japan). The graded lesions were subjectively classified as absent, mild, moderate, or severe according to lesion area [13] and the cellular infiltration as infiltrated cells equivalent to normal, poorly infiltrated cells, moderately infiltrated cells, and densely infiltrated cells [17].

### 2.11. Statistical Analysis

The data are expressed as mean ± SEM for each experimental group. The results were statistically analyzed using one-way analysis of variance (ANOVA) followed by Tukey's test. The software used was GraphPad Prism version 5.01, GraphPad Software, Inc. Differences were considered significant at *p* < 0.05.

## 3. Results

### 3.1. Cell Viability Analysis (MTT Assay)

Citral was the major component of the lemongrass essential oil (data not shown) and did not induce cytotoxicity in the cell viability assay. Citral at different concentrations of 3, 10, 30, and 90 *μ*/mL presented cell viability of 95.53, 86.52, 81.10, and 96.87%, respectively, indicating that citral did not induce cytotoxicity in any tested concentrations.

### 3.2. Radical Scavenging Assay

**Figure 1:**
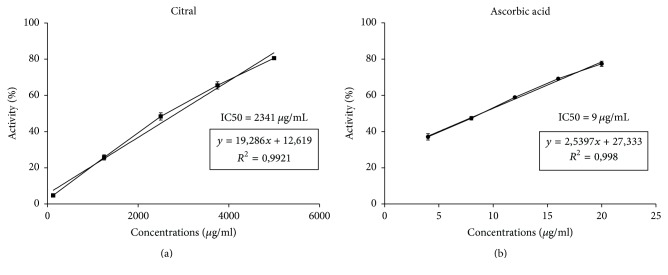
Antioxidant activity of citral. The figure shows the percentage of neutralization of DPPH by (a) citral and (b) ascorbic acid in the DPPH assay (*μ*g/mL).

The DPPH radical scavenging activity of citral was evaluated spectrophotometrically at doses of 125–5000 *μ*g/mL which showed antioxidant activity in vitro (IC50 = 2341 ± 0.07 *μ*g/mL). Ascorbic acid (positive control) scavenged DPPH radicals completely, and its IC50 value was 9 *μ*g/mL (Figure 1).

### 3.3. Effect of Citral on Serum Transaminases and Phosphatases Activity from APAP-Induced Hepatotoxicity

**Figure 2:**
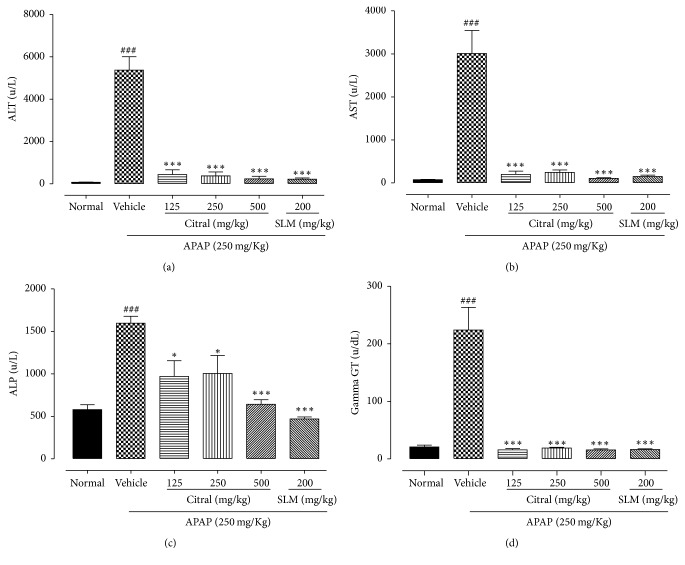
Effects of citral and SLM against APAP-induced liver toxicity in biomarkers of hepatic damage. Serum ALT (a); AST (b); ALP (c); and *γ*GT (d) enzyme levels. Values are mean ± SEM. ^###^*p* < 0.001 versus normal group. ^*∗*^*p* < 0.05 versus APAP group; ^*∗∗∗*^*p* < 0.001 versus APAP group.

In order to assess the effect of the pretreatment with citral a serum analysis of ALT, AST, ALP, and *γ*GT was performed (Figure 2). Administering APAP to the mice caused significant (*p* < 0.001) liver damage and necrosis of cells as evidenced by the elevated serum hepatic enzymes ALT, AST, ALP, and *γ*GT compared with normal group. Conversely, effects of pretreatment with different doses of citral (125, 250, and 500 mg/kg) exhibited a significant (*p* < 0.05) decrease in serum activities of ALT (91.79%, 93.07%, and 95.61%, resp.), AST (93.40%, 91.89%, and 96.52%, resp.), ALP (39.29%, 37.07%, and 59.80%, resp.), and *γ*GT (92.83%, 91.59%, and 93.0%, resp.), when compared to the APAP group. Similar results were found in pretreatment with SLM on the activity of ALT (95.90%), AST (95.03%), ALP (70.52%), and *γ*GT (92.69%).

### 3.4. Citral Reduces Leukocytes Chemotaxis In Vitro

**Figure 3:**
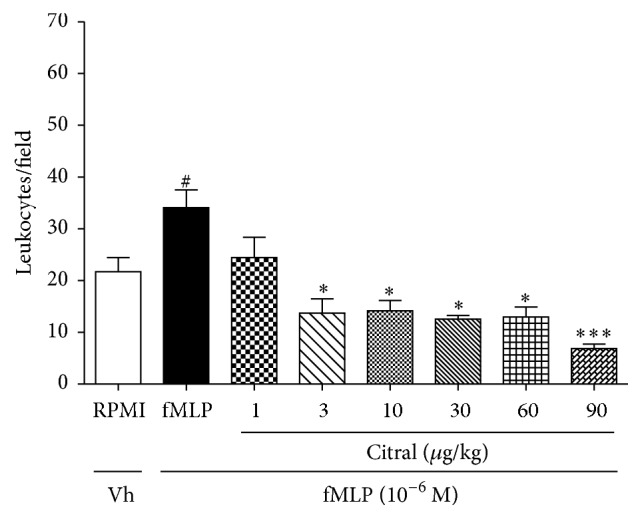
Effect of citral on in vitro leukocyte chemotaxis. Leukocytes were obtained from zymosan-induced peritonitis (1 mg/cavity) and stimulated with fMLP (10^−6 ^M) 30 min after citral treatments at doses of 1, 3, 10, 30, 60, and 90 *μ*g/mL. Values are mean ± SEM and are representative of three independent experiments. ^#^*p* < 0.05 versus RPMI; ^*∗*^*p* < 0.05 and ^*∗∗∗*^*p* < 0.001 versus group of leukocytes stimulated with fMLP.

To investigate the effect of citral on leukocyte migration, in vitro chemotaxis assay was performed (Figure 3). fMLP induced a significant leukocyte migration (*p* < 0.001) when compared with the control group (RPMI 1640). Citral significantly reduced neutrophil migration toward fMLP (10^−6 ^M) at doses of 3, 10, 30, 60, and 90 *μ*g/mL (34.06 ± 3.44, 13.70 ± 2.77, 14.15 ± 2.00, 12.55 ± 1.93, and 6.85 ± 0.89, resp.).

### 3.5. Citral Decreased MPO Activity and NO Production

**Figure 4:**
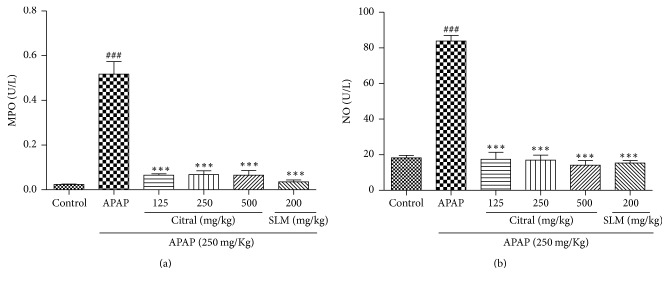
Effect of citral and SLM on (a) myeloperoxidase activity and (b) nitric oxide production. Values are mean ± SEM (*n* = 5). ^###^*p* < 0.001 versus animals control groups. ^*∗∗∗*^*p* < 0.001 citral or SLM pretreated groups versus APAP group.

The APAP overdose increased significantly MPO activity compared with normal group (Figure 4(a)). Moreover, the MPO activity was significantly reduced at all doses (125, 250, and 500 mg/kg) in citral-pretreated mice (87.41%, 86.87%, and 87.45%, resp.), compared with the group that received only APAP (*p* < 0.001). Conversely, NO concentration in liver tissue was decreased in the citral groups (79.17%, 79.80%, and 83.16%, resp.), when compared to the APAP group. The SLM group decreased considerably the MPO activity (93.09%) and NO concentration (81.78%) in the liver compared to APAP group. The results are shown in Figure 4(b).

### 3.6. Liver Histopathology

**Figure 5:**
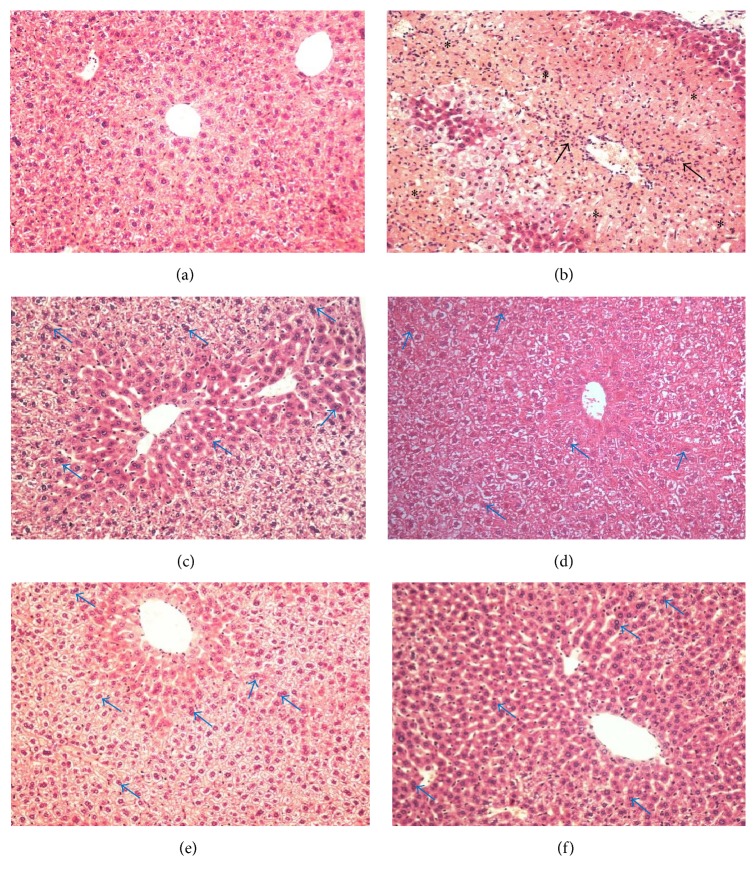
Effect of pretreatment with citral on the liver tissue morphology. (a) Control mice liver showed normal morphology and absent lesion area; (b) APAP group (mice liver that received orally APAP on last day of treatment, 250 mg/kg): presence of severe necrosis (*∗*) and inflammatory infiltrate (arrows); (c) group pretreated with SLM (200 mg/kg) + APAP; (d) 125 mg/kg citral + APAP; (e) 250 mg/kg citral + APAP; (f) 500 mg/kg citral + APAP. ((c)–(f)) Presence of binucleate hepatocytes (arrows in blue) and mild lesion area. Original magnification 20x. The sections stained with hematoxylin and eosin.

Histological sections of livers of normal mice showed a lobular architecture and hepatocytes normal structure (Figure 5(a)). On the other hand, severe necrotic areas were visible in the APAP-treated group, characterized by large areas of necrosis with centrilobular vein congestion, presence of a dense and/or moderate polymorphonuclear infiltrate, and vacuolization of hepatocytes (Figure 5(b)). In the SLM group, poorly infiltrated cells, preserved portal areas, some vacuolated cells, and mild injury in the centrilobular region were observed (Figure 5(c)). The severity of hepatic injury has improved with citral pretreatment (Figures 5(d) and 5(e)). Animal treated with citral showed mild injuries, whereas hepatic parenchyma without necrosis with centrilobular area preserved and poorly infiltrated cells. Furthermore, a mild vacuolization and the presence of binucleate hepatocyte were observed with citral pretreatment. Additionally, pretreatment with 500 mg/kg of citral showed the hepatic parenchyma similar morphology to the control group. The liver architecture was preserved with apparently normal hepatocytes, presence of binucleate hepatocytes, and the cellular infiltration equivalent to normal (Figure 5(f)).

## 4. Discussion

*Cymbopogon citratus*, commonly known as lemongrass, is widely distributed worldwide and is commonly used in traditional Indian, Chinese, and Brazilian medicines [18]. The essential oils from* C. citratus* contain various monoterpenes, including citral (mixture of neral and geranial), the most pharmacologically and physiologically important constituent [6, 19]. In previous study [11], we demonstrated that lemongrass essential oil improves the hepatic injury caused by APAP, and in this present study the effect of citral, the major component of this oil, was evaluated.

Natural products have important biological properties in disease prevention as in hepatoprotective capacity. This activity of natural products can be explained by its antioxidant properties deriving from monoterpenes, flavonoids, phenols, and so forth [20]. The DPPH assay has been widely used to determine the free radical scavenging capacity of various samples because of DPPH stability, simplicity, and fast assay [21]. In this assay, citral showed antioxidant properties due to the DPPH scavenging activity, as also observed in previous studies [22–24]. In general, the hepatoprotective activity of plants can be considered as an expression of the functional improvement of hepatocytes that results from accelerated cellular regeneration [13, 20, 25]. Therefore, SLM that has been employed as a protective treatment of liver disease by its antioxidant properties deriving from the phenolic nature of flavonolignans [13, 16, 26]. The MTT assay in vitro performed was used to evaluate the cell viability after citral treatment. The results showed that citral, even at a higher concentration, was absent from neutrophil toxicity. Our data were also observed by Bachiega and Sforcin [9], Bouzenna et al. [23], and Shen et al. [8].

In high doses of APAP, the oxidation pathway is initiated by the formation of the reactive metabolite NAPQI, which is generated mainly by the cytochrome P450 enzymes Cyp2e1 in mice and humans [3]. Excessive NAPQI formation after APAP overdose depletes cellular glutathione, adducts proteins, including mitochondrial proteins, and induces mitochondrial oxidant stress and dysfunction; this results in nuclear DNA fragmentation and necrotic cell death [27].

APAP overdose results in destruction of liver cells in turn resulting in the elevation in serum level of enzymes aminotransferases [28–30]. The measuring serum levels of specific liver enzymes such as ALT, AST, ALP, and *γ*GT are most commonly used markers in hepatotoxic studies. Therefore, serum hepatic biomarkers analysis is important for identification of liver lesion [16, 28, 30, 31]. In our work, APAP administration (250 mg/kg) caused acute liver injury in mice, characterized by an increase in serum activity of transaminase and phosphatases (AST, ALT, ALP, and *γ*GT). In addition, the toxic effects of APAP were also observed histologically, showing severe necrotic areas with centrilobular vein congestion, presence of inflammatory cell infiltration, and vacuolization of hepatocytes. In contrast, our results showed that pretreatment with citral was able to reduce levels of AST, ALT, ALP, and *γ*GT at all doses employed, improving liver damage when compared with the APAP-treated mice. The liver histopathological analysis in groups pretreated with citral showed hepatocytes preserved, infiltrated cells equivalent to normal, mild lesion areas, and the morphology of the hepatic parenchyma similar to the control group, suggesting a protective effect of citral. Different from our results, Li et al. [24] did not show a significant difference in the levels of AST and ALT among rats pretreated with citral compared with APAP group. However, in their work, the rats were fed a pelleted laboratory diet with a single dose of citral and the APAP was given intraperitoneally. This difference in the experimental design between the work of Li et al. [24] and our study may have contributed to the difference in AST and ALT results.

In APAP hepatotoxicity, the oxidant stress results in nuclear DNA fragmentation followed by an inflammatory response, which includes the release of proinflammatory cytokines and the activation of immune cells [2, 27]. Neutrophils and macrophages can also potentially aggravate the injury, as well established in hepatic ischemia reperfusion injury and obstructive cholestasis [32, 33]. In the present study, neutrophil migration was measured indirectly by MPO activity and citral pretreatment decreased the MPO activity compared to the group APAP, suggesting decrease of neutrophil infiltration. These findings were consistent with the reports by Shen et al. [8], in which citral treatment decreased significantly MPO activity induced by LPS. Moreover, in our study of the in vitro chemotaxis assay, fMLP was used as a chemotactic agent which promoted neutrophil migration and citral reduced the leukocyte migration, suggesting an anti-inflammatory activity of this monoterpene. These results were also correlated with a discrete lymphocytic infiltrate observed in citral-pretreated group obtained by histopathological observation of the liver.

NO exerts important roles as mediator of toxicity of APAP in the liver. NO is a well-established marker of inflammation, and inhibition of its production can be a useful therapeutic strategy in inflammatory diseases [10, 34, 35]. Additionally, other inflammatory mediators, such as TNF-*α*, IL-1*β*, and IL-6, are associated with the severity of hepatic injury [8, 36, 37]. Several studies demonstrated that citral inhibits the release of important inflammatory mediators, such as TNF-a, IL-1*β*, IL-6, and NO [8, 9, 37–39], probably by inhibition of iNOS expression, NO production, and various LPS-induced pathways, including p38 mitogen-activated protein kinase (MAPK), c-jun NH2-terminal kinase 1/2, and the transcription factor NF-kB, as demonstrated by Lee and collaborators [7, 37]. In our work, the pretreatment with citral decreased the NO production, suggesting an anti-inflammatory activity of this compound.

Therefore, in this study, we demonstrated that citral has antioxidant activity. The pretreatment with citral prevented hepatic alterations caused by APAP acute toxicity, preventing the increase of hepatic injury markers (ALT, AST, ALP, and *γ*GT), MPO activity, and NO concentration in hepatic tissues. Some studies have shown the hepatoprotective effect of lemongrass on hepatotoxicity in experimental model in rats or mice [11, 40, 41] and according to our results, we suggest that this improvement in liver damage by pretreatment with lemongrass essential oil may be associated with citral content, since citral is the major constituent of* C. citratus *essential oil.

## 5. Conclusion

In conclusion, pretreatment with citral showed hepatoprotective effects on hepatic lesions caused by APAP overdose. This effect may be associated with the reduction of oxidative stress or have an influence on inflammatory events. Therefore, further studies are necessary to investigate the mechanism by which citral is metabolized to protect the liver.
